# Protein sequence analysis in the context of drug repurposing

**DOI:** 10.1186/s12911-024-02531-1

**Published:** 2024-05-13

**Authors:** Natalia García Sánchez, Esther Ugarte Carro, Lucía Prieto-Santamaría, Alejandro Rodríguez-González

**Affiliations:** 1 Centro de Tecnología Biomédica, Universidad Politécnica de Madrid, Pozuelo de Alarcón, Madrid, 28223 Spain; 2 ETS de Ingenieros Informáticos, Universidad Politécnica de Madrid, Boadilla del Monte, Madrid, 28660 Spain

**Keywords:** Drug repurposing, Sequence analysis, Protein sequences, Embedding vectors

## Abstract

**Motivation:**

Drug repurposing speeds up the development of new treatments, being less costly, risky, and time consuming than de novo drug discovery. There are numerous biological elements that contribute to the development of diseases and, as a result, to the repurposing of drugs.

**Methods:**

In this article, we analysed the potential role of protein sequences in drug repurposing scenarios. For this purpose, we embedded the protein sequences by performing four state of the art methods and validated their capacity to encapsulate essential biological information through visualization. Then, we compared the differences in sequence distance between protein-drug target pairs of drug repurposing and non - drug repurposing data. Thus, we were able to uncover patterns that define protein sequences in repurposing cases.

**Results:**

We found statistically significant sequence distance differences between protein pairs in the repurposing data and the rest of protein pairs in non-repurposing data. In this manner, we verified the potential of using numerical representations of sequences to generate repurposing hypotheses in the future.

**Supplementary Information:**

The online version contains supplementary material available at 10.1186/s12911-024-02531-1.

## Introduction

In April 2003, the Human Genome Project [1] completed the sequencing of the human genome essentially in its entirety. This paved the way for accelerating the study of human biology by employing gene and protein sequences. Sequence data presents important biological properties [2], which can be condensed in bioinformatic representation techniques such as simple or multiple sequence alignment, or learned in numerical vectors by Machine Learning (ML) encoding [3].

Encoding the sequences makes them eligible for further analysis and new drug repurposing (DR) approaches. DR consists in finding new uses for existing drugs [4]. This approach has emerged as an alternative to de novo drug development, which involves high costs, long times, and risks in research. Pharmacological candidates for DR can be found in diseases that share the same molecular relationships and phenotypical manifestations [2]. Furthermore, we assumed that if certain properties and similarities between biological sequences can be captured, they can also convey functional information across pathway, disease, drug, and target network knowledge. However, the potential of sequence sensitive-guided drug repurposing has not yet been broadly explored [5]. For that reason, in this article, we worked towards demonstrating the importance of the protein sequences that participate in known successful DR cases.

The present study has been developed in the context of DISNET project [6]. DISNET is based on the Human Disease Network (HDN) concepts [7]. The purpose of this project is to exploit similarities in biological, pharmacological, and phenotypical characteristics among diseases to uncover knowledge and patterns that would otherwise be left unnoticed and generate novel DR hypotheses [8, 9]. To achieve this goal, the project has developed a complex multilayered network that integrates heterogeneous biomedical information on diseases and their relationships.

The principal objective of the current research was to analyse the role of protein sequence data in DR. As a secondary objective, we reviewed different protein sequence representation methods and investigated their relevance to protein comparison, particularly in the context of DR. We aimed to discover whether general encoding strategies were able to encapsulate important features that take part in known DR cases. For that purpose, we examined significant differences between pairs of proteins participating in DR cases and those not participating in DR cases.

The manuscript is structured as follows. State of the art section reviews drug repurposing approaches based on biological sequence data and different encoding methods. Materials and methods section explains the materials and methods used for protein sequence data analysis, Results and discussion section shows and discusses the results obtained, and Conclusions section describes the conclusions and future lines.

## State of the art

Drug repurposing (DR) approaches have reduced costs, risks, and time for drug development compared to traditional drug discovery methods [4]. As a result, they have provided promising solutions in cases where research is lacking investment or time. Examples of this include drug development for pandemics like COVID-19 [5], or genetic rare diseases [10, 11].

Computational methods for DR continuously aim to narrow down the scope of the chemical search to find old drugs as new candidate treatments. This accelerates the research and clinical trials of candidate drugs [12]. A potentially enriching source of information guiding DR are biological sequences. Next Generation Sequencing (NGS) and high-throughput technologies [13] have led to a boom in unlabelled biological sequence data that can be carefully exploited for computational drug discovery and repurposing [14, 15]. However, manually curated data from these databases is scarce in comparison with sparsely annotated databases, and creating an annotation gap can benefit from advances in biological sequence representation learning [15]. Furthermore, the emergence of large-scale data has generated more interest in efficient data-driven approaches rather than high-precision property-based experimental data methods that are more time-consuming [16].

Nevertheless, it is important to note that providing representations that capture patterns and biological properties to machines is also vitally important. The first step towards achieving this objective is passing the sequences into numerical values or encoding, starting with finding the atomic unit of information (referred to as sequence tokens) [17, 18] that effectively defines fragments of biological sequences. This can be done either by capturing them in nodes in a graph or with condensed elements in a 1D representation [19]. In this line of work, we will be focusing on vector representation learning. There are two types of methods: (i) direct encoding creates a vector of numerical features for each position or token in a sequence, whereas (ii) indirect encoding works with an established number of features for the encoded sequences [20]. These methods can in turn be separated into four different encoding strategies that place more emphasis on sequence or token similarity in a sequence: binary encoding, evolution-based encoding, property-based encoding, and machine learning encoding.

- **Binary encoding**

One of the most extended strategies, often used as input for ML models [21, 22], is to encode characters (nucleotides or amino acids) in a sequence as categorical binary encoded variables. In other words, n-bit vectors are formed for each position of the sequence with information about the occurrence of each possible n sequence character in the set of sequence alphabets (single or supergroup characters). They do so as hot encoded elements (receives 1 for the presence of a character in a set while the rest of the characters are fixed to 0, e.g., Adenosine in DNA is [1, 0, 0, 0]). The main limitation of this model is the increase in dimensionality involved in larger character sets or longer sequence lengths, for instance, in proteins [23].

- **Evolution-based encoding**

The fact that new sequences are adapted from preexisting ones allows us to model sequences using probabilistic models on evolutionary preference of sequence data across time. Some probabilistic representation methods include taking descriptors of similarity characteristics based on amino acid substitution probability in the PAM and BLOSUM matrices [24]. Other methods have been based on multiple sequence Position Frequency Matrix (PFM) encoding, which takes the frequencies of biological “characters” at each sequence position to construct Position Specific Scoring Matrixes (PSSM) as feature descriptors applied to improve the performance of various predictors of gene and protein attributes [25, 26]. In position-dependent methods, PSSM profiles are found, generally in fast profile iterated alignment algorithms such as Position Specific Iterated BLAST (PSI-BLAST) [27] or Hidden Markov Models (HMM) models [28]. However, alignment is increasingly time-costly and unable to learn remote homology and patterns from large-scale databases in this post-genome era [14]. To this day, only two [5] freely available comprehensive web-based resources have implemented alignment sequence-guided repurposing tools: DrugBank [29, 30] and NOD [5].

- **Property-based encoding**

It is also helpful to convey domain knowledge about biological and physicochemical features associated with sequence data representations as well. For instance, physicochemical characteristics like hydrophobicity, ionization, and solubility, play a critical role in proteins’ functions and structure formation, and physicochemical characteristics such as secondary structures, Van der Waal’s interactions and hydrogen bonding have been taken into account in categorical data encoding in amino acids [31] and RNA [32]. Distance patterns between amino acids can also carry important structural information. For instance, [33] created a state-of-the-art directed - graph based representation of distances between characters in a sequence set.

- **Machine learning encoding**

Simple encoding methods often do not capture the vital context to understand the structure of contact patterns in biological sequences, especially proteins [17]. Frequently, as a post-encoding stage, ML encoding methods cover statistical sequence modeling to capture Probability Distributions (PD) and complex context dependencies between biological characters in scalable-sized sequence corpuses [16]. To this end, this subfield of ML and linguistics called Natural Language Processing (NLP) applies Language Models (LM), or probabilistic learning, to fragmented sequences composed of token vectors of real value, generally passing them to Neural Network (NN) model architectures [3, 15, 18]. As a subset of ML, an advantage of Deep Learning (DL) is that it can learn task-related representations from non-linear, noisy, and high-dimensional input like sequences, and generally create fixed output embeddings with dimensions determined by the last hidden layer number within the network. These probabilistic approaches provide the basis for the NLP distribution hypothesis: “similar words or sequences are often given in contexts of similar meaning or function”. In this way, NLP aims to reveal life functions and biophysical constraints encoded in “the language of life” [18].

## Materials and methods

Protein sequences are eligible candidates for the elucidation of molecular interactions and disease relationships, thus providing plausible information for the generation of DR hypotheses. To analyse the role of these biological sequences in DR scenarios, we followed the subsequent procedure. In Data acquisition and integration section, we describe the employed data registered in DISNET, as well as the sources accessed in its extraction. Moreover, we present the DR successful cases that were gathered. Embedding sequence data to numerical vectors section provides methods for the encoding of protein sequence data and Visualization of the embeddings section for its visualisation. Eventually, in Analyzing sequence data in successful DR cases section, we explain the creation of a strategy framework to integrate the different embedding approaches as a basis for DR. We sought to validate this methodology by performing statistical tests between the DR proteins and the rest of the DISNET proteins.

All the data we employed within the research can be found in the file “data.xlsx” (https://medal.ctb.upm.es/internal/gitlab/disnet/sequences/embeddings-in-dr).

### Data acquisition and integration

DISNET database incorporates biomedical knowledge by mining and querying heterogeneous public sources [6]. It integrates data regarding diseases such as related symptoms, genes, proteins, drugs, or drug targets. This information is structured in three levels: the phenotypical layer (with mainly disease-symptom associations), the biological layer (with the associations of diseases to genes and proteins, among others), and the drug layer (with drug-related data, including their associations to diseases and their targets).

As the main objective of the present study is to analyse whether or not protein sequences play a significant role in DR processes, we worked with DISNET platform data. In this manner, we considered the protein sequences, proteins, genes, diseases, drugs, drug targets, and their associations. In the Supplementary Materials (SM), SM Table 1 shows the details of the data typology.

### Embedding sequence data to numerical vectors

Out of the protein sequences in the DISNET database, we generated several illustrative types of representation vectors within the four different encoding strategies defined in State of the art section.

- As a naïve baseline method, we obtained One-hot encoding vectors.
- Furthermore, to exploit a distance-based encoding method, we performed a graph representation vector encoding called Sequence Graph Transform (SGT) [33]. This method aims to represent global distance patterns between character sets of a sequence as if they were distances present in a directed Graph Representation. To do this, it applies a non-linear transform function on the set of amino acid distances.
- Finally, we collected a variety of pre-trained NLP word embeddings and probabilistic language models to predict protein embeddings, among which the BioVec [34], word2vec [35], ProtBERT [36] unsupervised, ESM-1b [37], and SeqVec [34] and Bepler [33] models were tested.

One-hot and NLP protein encodings were performed with the Python embed module “bio_embeddings” package, which provides the pipeline and encoders for reproducible embedding generation [38]. SGT distance encodings were carried out using the open source code available from its original paper [33]. For this analysis, the embedding methods producing tensors with>1 dimensional features instead of regular feature vectors were reduced. This was undertaken with a mean pooling method implemented in PyTorch, by using the ‘bio_embeddings_reduce_embeddings” class module. This method calculates the means of the hyperparameter features for every element associated with it.

In addition, we needed to pre-process the sequence data. Protein sequences in the database ranged from 16 to 34,350 amino acids in length. To reduce general computation in all models and filter out sequences that were less representative and had less learning capacity in pre-trained models, we have removed proteins with more than 6,024 amino acids. Of 18,520 proteins, 13 proteins were discarded, except for those in the SGT model, which presented lower computation demands, so the total corpus of 18,520 proteins was encoded.

In order to select the most informative embedding methods for our analysis, visualizations of the embeddings in a low dimensional map were done as is explained in the following subsection. We selected the unsupervised methods that kept data more aggregated into informative clusters. In other words, we generated lower-dimension vectors (2D and 3D) with the computed embeddings to graphically represent whether the protein groupings in the plane of the 3D space were biologically representative. After this, we computed the distances between the embeddings of all protein pairs in the dataset using the cosine distance, as we will describe in Analyzing sequence data in successful DR cases section. Methods that produce a wider distribution of cosine distances were found to have a higher sensitivity to differences in the feature vectors.

Table 1 describes the final selected methods implemented in this line of work. Two methods were based on encoding and two on ML algorithms. A more extensive review of the total embeddings that were tested and their applications can be found in SM Table 2.

**Table 1:** Characteristics of the final selected embedding methods. Rows describe the method (strategy of encoding used and established in Embedding sequence data to numerical vectors section), the input and output (data types used as input and output for all methods), and the embedding feature size (dimensions of final numeric representations). A description of the model architectures and the open code libraries used for embedding generation is also given. Some models provide pre-trained models. Databases of protein sequences used in these cases are given as well

	One-Hot Encoder	Sequence Graph Transform	SeqVec	BERT ProTrans
Method	Binary encoding	Distance encoding	DL bidirectional contextual LM	DL masked contextual LM
Input	Sequence token list	Sequence token list	Encoded token list	Encoded token list
Output	1 Tensor	1 Feature vector	3 Tensors (1 per layer)	1 Tensor
Embedding feature vector size	(sequence length x 21)	441	(sequence length x 1024)	(sequence length x 1024)
Implementation libraries	-	sgt package	Pytorch / AllenNLP	Pytorch / Tensorflow
Architecture	-	-	biLSTM (Recurrent RNN)	Transformer
Layers (nodes)	-	-	1 CNN (1024) + 2 biLSTM (1024 nodes each)	30 layers of biLSTM stacked encoders
Supervision	-	-	-	Available on structural localization supervision
Pretrained models	-	-	Yes	Yes
Databases used in pretraining			UniRef50 (33M sequences)	Big Fat Database (BFD)

### Visualization of the embeddings

One of the fundamental steps of encoding biological sequences is to validate if the vector representations previously learnt constitute a biological context on their own [15, 38]. In this regard, it is of importance to assess how well the unsupervised language models consistently discriminate embeddings across functionally relevant properties in the DISNET database. For this purpose, visualizations of the protein embedding space were performed for proteins having a unique functional protein class. Both the described classes and the identifiers belonging to the classes were extracted from PANTHERdb [39], based on the classification of the complete evolutional and functionally related protein families. For a plausible and informative visualization, only proteins associated exclusively with one functional class were collected.

Moreover, it was necessary to project the multivariate feature vectors into two or three dimensions to graphically display the visualization of the protein space with the class mappings. To do this, three methods of dimensionality reduction were explored. These methods preserve as much information as possible about the structure present in high-dimensional data in a low-dimensional visualizable map.

We employed T-distributed Stochastic Neighbour Embedding (t-SNE) dimensionality reduction, used for multidimensional feature data that relate in a non-linear way [40]. It is a variation of its predecessor Stochastic Neighbor Embedding (SNE) and is used in a variety of machine learning methods because of its easier optimization and quality visualization. It captures the multidimensional data similarity structure into a reduced manifold representation by creating a single map that reveals the same distance probabilities between data points from the original data in the reduced data manifold.

### Analyzing sequence data in successful DR cases

**Fig. 1:**
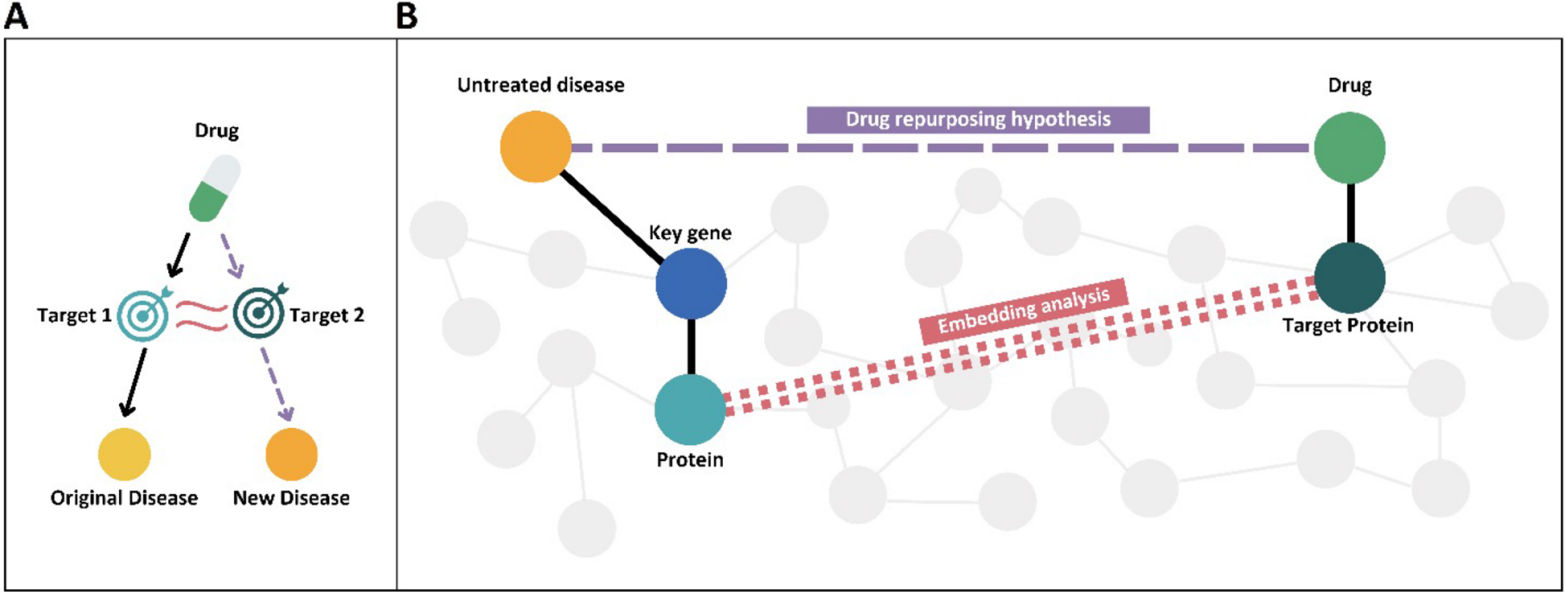
Sequence drug repurposing approach. **A** The idea in which protein similarity between two proteins Target 1 and Target 2 can generate new prescriptions from a drug from an old disease to a new disease. **B** A schema of the framework that presents the proteins put forward for similarity search against drug targets, which are those encoded by key genes associated to diseases without treatment

The final objective of this work is to evaluate whether the use of protein sequence data with the previously described methods can be truly informative or not. The main approach plausible for DR hypotheses generation based on sequences is the one regarding target sequence similarity. That is, proteins associated with diseases in the database, which share similarity to a target already targeted by another drug, could be a plausible subject for therapeutic repurposing (Fig. 1A). This is assumed on the basis that part of the identity shared by the two proteins will have underlying drug - target binding regions or similar functions that in turn affect downstream pathways causing the manifestation of the two diseases. In this manner, the future approach would be to compare the distance of the embeddings or the significance metrics of the alignments of proteins related to untreated diseases against drug targets (Fig. 1B).

**Fig. 2:**
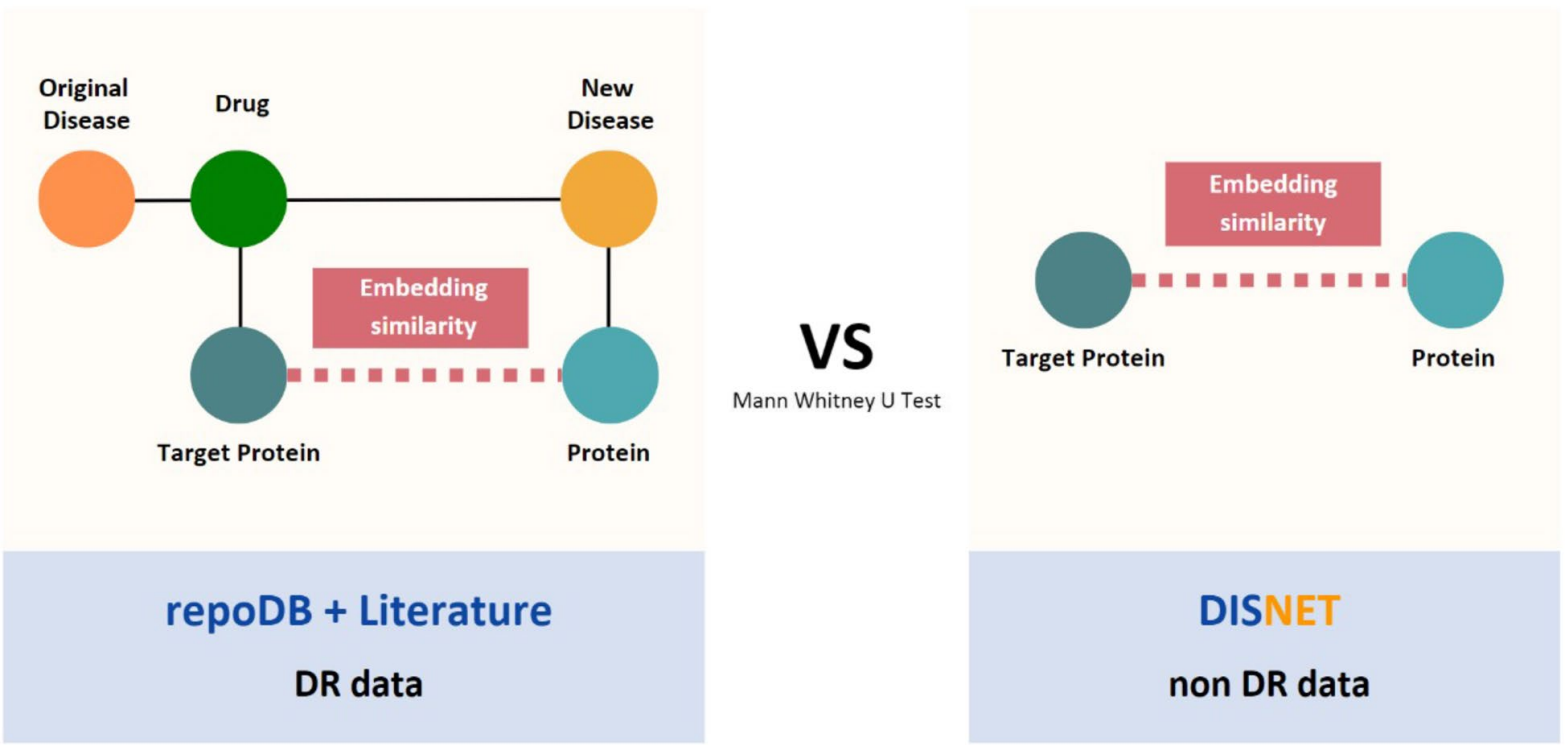
We calculated the similarity of the “drug target protein - protein” pairs embeddings corresponding to the DR data (repoDB and Literature) and compare it to the protein pairs of the non-DR data (the rest of DISNET) through a Mann Whitney U Test. This procedure was done for the “Filter by class” subset too

Consequently, it was necessary to determine whether the similarity metrics taken from the global DISNET subsets were significantly different from those taken with protein-target pairs from the real DR cases data. We conducted a statistical signification test that compares distance metrics between proteins that participate in DR successful cases and the rest of possible combinations of proteins in the DISNET knowledge base (Fig. 2). To this end, a Mann Whitney U Test (MWU) was carried out. Non-parametric Mann Whitney U tests are used over samples lacking normal distributions and with varying sizes and variances to test the hypothesis that two populations have equal medians [41]. To tackle the large differing population sample sizes between total and repurposed protein pair distance data, 1000 MWU tests were performed with random samplings from the total protein pair distances. This way, the two compared samples (DR vs the rest) had similar sizes and we reported a distribution of p-values. More details on this matter are provided in the Supplementary Material section.

The DR cases were taken from 2 different sources. On the one hand, some cases were taken from repoDB [42]. RepoDB is a database comprised of successful and failed indications for drugs gathered from DrugCentral [43], and ClinicalTrials.gov. On the other hand, other cases were collected from the scientific literature and mapped to DISNET’s vocabularies, as described in Prieto Santamaría et. al. [8]. We only took into account DR information relative to indications of new diseases. That is, the diseases for which the drugs were repositioned and not originally indicated. This dataset is denominated throughout the study as *Literature*. In both sources, we removed the cases where the disease and the drug shared the drug target protein as they were not relevant for the present study. We needed cases where the repurposing cause was not the target on which the drug acts, in order to analyze the role played by the remaining proteins in the DR processes.

Out of these two datasets, we generated an extra subset of the protein DR cases for each dataset, denominated “Filtered by protein class”. It was particularly limited to protein-target pairs that did not share the same PANTHERdb protein class [39]. This exclusion was done under the assumption that proteins presumably belonging to the same family would give high similarity results but would not offer alternatives for repurposing, meaning that the implicit protein relatedness would not yield novel insight in the DR context. The same statistical process and testing was performed with this subset of DR data. The number of pairs tested involved in each subset is shown in Table 2.

**Table 2:** Description of DR case subsets and rest of possible combinations of proteins in DISNET. The total number of possible combinations of protein pairs is 171,152,751

Source of DR info	Subset	Number of protein pairs	
		Unfiltered	Filtered
RepoDB	Repurposing	182,867	51
	DISNET	171,134,829	171,152,700
Literature	Repurposing	93,835	43
	DISNET	171,058,926	171,152,708

Cosine distances were calculated for all combinations of protein target pairs present in the DR cases. It measures the similarity between two reduced protein embeddings, as it computes the cosine of the angle between two vectors projected in a multidimensional space [44]. In this way, it succeeds in reflecting a relative comparison between protein encodings. It is calculated by obtaining the angle 0 by the scalar product and the norm of *a* and *b* (Eq. 1).

1 $$\begin{aligned} cos(\theta ) = \frac{a \times b}{\Vert a\Vert \times \Vert b\Vert } \end{aligned}$$

It is important to here mention that we have two types of vectors depending on the embedding methods generating them:

- Those generated from OneHot and SGT, whose values across all dimensions have positive values. This leads to cosine similarity $cos(\theta )$ to range from 0 (most distant pairs) to 1 (most similar pairs).
- Those generated from NLP-like methods (SeqVec and ProtBERT), whose values in some of the vector dimensions can be negative. This leads to cosine similarity $cos(\theta )$ to range from -1 (completely opposite vectors) to 1 (same vectors).

Along the manuscript, we refer to the cosine distance as the inverse of the presented Eq. 1, that is, $distance = 1 - cos(\theta )$. Therefore, this distance will vary from 0 (most similar pairs) to 1 (most distant pairs) in the vectors corresponding to the first point (OneHot and SGT). But for the second ones (SeqVec and ProtBERT) will vary from values of 0 (most similar pairs) to 2 (most distant pairs).

## Results and discussion

The main goal of the present work was to analyse the role of sequence data in successful DR cases and DISNET data to validate its informative content for a future generation of new DR hypotheses. Prior to examining protein sequences, different methods of sequence embedding were explored. The final four methods in which we have focused were OneHot reduced, SGT, BERT reduced, and SeqVec reduced. Their respective vector dimensions were: 21, 441, 1024 and 1024. DL based contextual learning methods have higher dimensions involved, corresponding to the hyperparameter of the last layer of hidden nodes.

To graphically represent the encodings, three dimensionality reductions were performed across PANTHERdb distinct functional classes and disease association count intervals. Finally, the aggregation of proteins across these properties was studied to test if the resulting protein encodings were biologically representative. The resulting dimensionality reductions showed general aggregation for protein functional class labels. It is important to note that a pooling method was undertaken to representations of the hidden layer output in the NLP embeddings to encoded vectors in one dimension for all methods. This approach is known to be suboptimal [15].

**Fig. 3:**
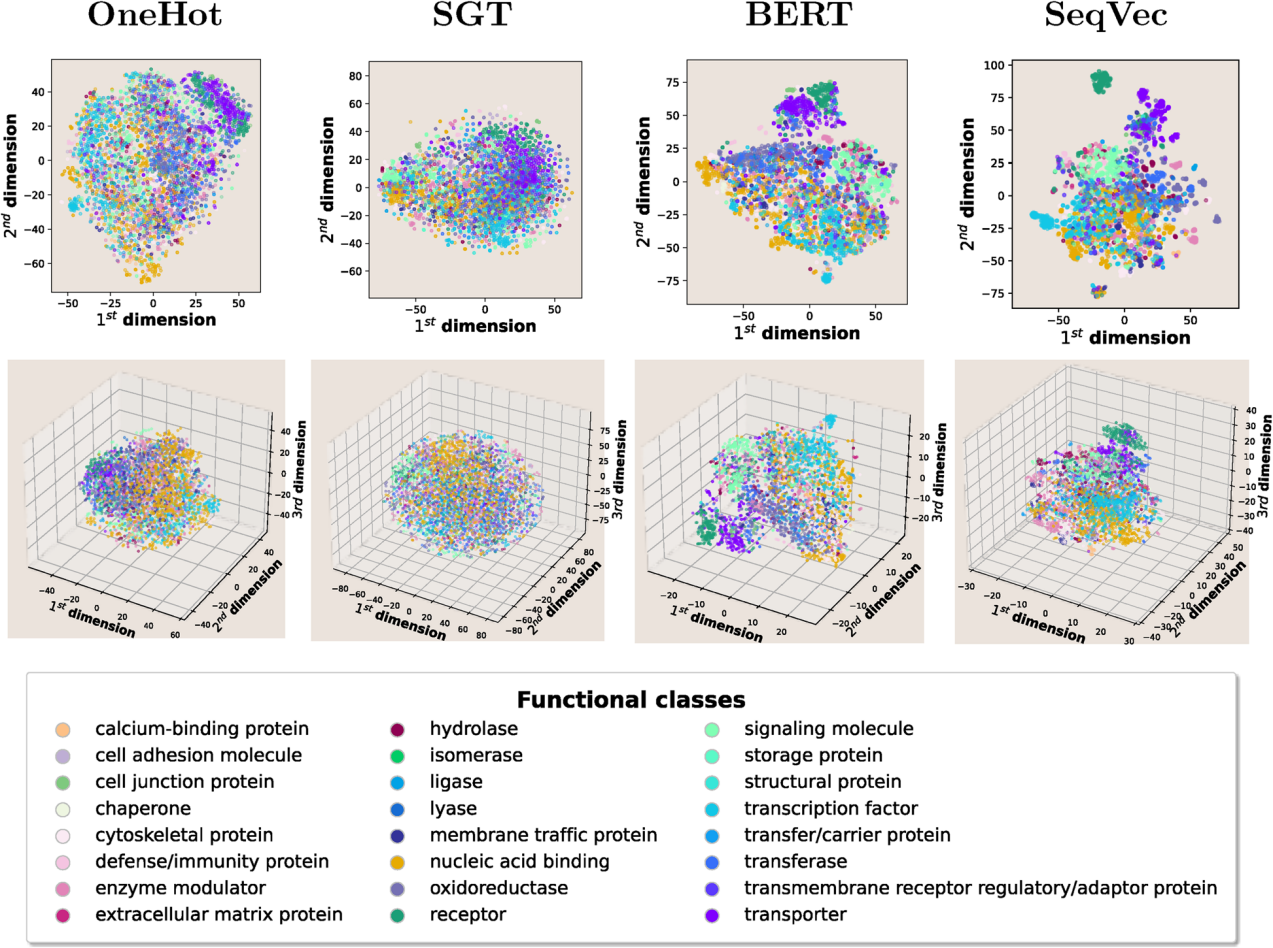
An example of 2D and 3D T-SNE dimensionality reduction plots of the protein sequence embeddings, coloured by functional class. Each reduced protein encoding belonging exclusively to a functional class is scattered in the 2D, 3D protein space and plotted in the graph. The colour of each protein represents its PANTHERdb class

Moreover, it is important to highlight the difference between the reductions of the encoding methods. The pretrained DL models based on sequence contextual learning SeqVec and ProtBERT deliver significantly better results. This is possibly because of the higher number of dimensions present in their resulting embeddings, as well as the fact that they can better encapsulate protein patterns by transferring learning from large protein corpuses. An example of a functional class visualization with T-SNE reductions across all methods can be found in Fig. 3.

To validate the use of protein sequence data for the future detection of DR candidates, a target similarity search was performed at the embedding level. We compared the distances between proteins that participate in successful DR cases and the rest of possible combinations of proteins in the DISNET knowledge base.

We analyzed successful DR data. To do this, proteins highly associated to the diseases present in DR cases were gathered. These proteins were screened for similarity against targets of drugs present in cases of DR. In parallel, a similarity screening was performed for the rest of the protein pair combinations in DISNET. To this end, the cosine distances and alignments were computed for the mentioned pairs. Moreover, we distinguished RepoDB and Literature data from data resulting from filtering those cases in which the two proteins belonged to or shared the same protein class.

All the data regarding these cosine distances between proteins based on the embedded vectors and in the named different subsets can be found at the Supplementary Material “data.xlsx” file. For the sake of understanding, we here include one example of a DR case and its comprising elements. As previously depicted in Fig. 2, DR data consists of two proteins: one of them is the target of the drug that has been repurposed; and, the other one is related to the new indication to which the drug has been repurposed for. For example, the drug “celecoxib” was repurposed to treat the disease “Rheumatoid Arthritis” (this association has been stated both in the Literature and in RepoDB). Celecoxib targets the protein P35354 (encoded by PTGS2 gene, named “prostaglandin-endoperoxide synthase 2”) and Rheumatoid Arthritis is associated with the protein Q92743 (encoded by HTRA1 gene, named “HtrA serine peptidase 1”). These two proteins show a cosine distance of 0.06 between OneHot encoded vectors, 0.17 between SGT encoded vectors, 0.30 between ProtBERT encoded vectors, and 0.42 between SeqVec encoded vectors. All of these distance values are below the average distance of each method encoded pair of vectors, meaning that the two proteins in this DR case are closer than expected.

For 37 DR cases present in RepoDB and/or in the Literature (out of a total of 67), the distance between the protein pairs has yielded lower values than the mean for the 4 different embedding methods. In other words, the 4 studied embedding techniques provided protein vectors whose distance was lower than the mean for 55,22% of these protein pairs. For the rest of protein pairs, the distance was below the mean for at least 2 of the embedded vector pairs. None of the pairs generated for the 4 embedding methods distance values above the mean. This fact highlights the ability of the embedded vectors of representing repurposing information, since they place proteins participating in DR scenarios closer in the space. It would be interesting and we hypothesise that these mean distance values could be used in the future as thresholds when generating new repurposing hypothesis based on sequences. Some examples are provided later in the document.

A table describing the number of drugs, diseases, and protein-target pairs implicated in the vector similarity can be found in SM Table 3. The probability distributions of the cosine distances obtained by the different embedding methods are represented in SM Fig. 2.

**Fig. 4:**
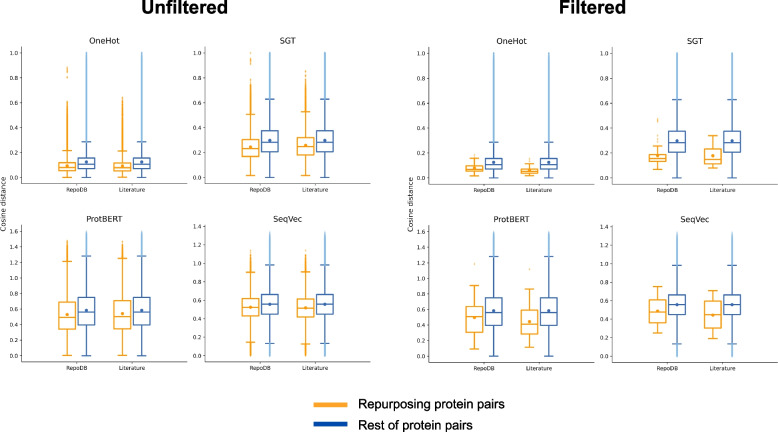
Boxplots comparing cosine distance distribution across the different subsets of protein pairs. We distinguish Unfiltered (left) and Filtered (right) subsets by same protein class. The distributions are shown for all embedding methods (OneHot, SGT, ProtBERT and SeqVec, depicted in each subplot) and sources of DR protein pairs (RepoDB and Literature, compared in the X axes). The main comparison is represented in two colours (orange for the repurposing protein pairs and blue for the rest of DISNET protein pairs). In each boxplot, central dots represent the mean value of the cosine distance and horizontal lines represent the median value of the cosine distance. For the sake of clarity, the annotations of the statistical tests comparing the medians are not represented since all differences between the compared subsets were statistically significant (but more information on this matter can be obtained in the supplementary materials). Proteins participating in repurposing known cases tend to be closer than the rest of pairs

As for the embedding testing, a compilation of the p-values obtained via Mann Whitney U statistical test comparing DR vs the rest of protein - pairs across all methods and sources can be found in Table 3. Statistical significance has been thoroughly evaluated through Mann Whitney U tests through sampling iterations in order to overcome the issue of differing sample sizes of compared distributions, and results are presented in SM Table 5 and SM Table 6. The results confirm the hypothesis testing that the median embedding distances in the DR population deviate significantly from those of the remaining DISNET population, showing less distance in the repurposing pairs. These findings lead to the conclusion that protein - target pairs in DR data are significantly and consistently more similar than global protein - target pairs in DISNET database. The comparison of the cosine distance distributions between DR and DISNET is shown in Fig. 4.

**Table 3:** Description of DR case subsets and rest of possible combinations of proteins in DISNET. The total number of possible combinations of protein pairs is 171,152,751

Source of DR information	Subset	DR vs DISNET cosine distance protein pairs p-value (mean ± standard deviation)			
		OneHot	SGT	ProtBERT	SeqVec
RepoDB	Unfiltered	0.00 (0.0947 ± 0.0597)	0.00 (0.2446 ± 0.1055)	0.00 (0.5288 ± 0.2396)	0.00 (0.5246 ± 0.1415)
	Filtered	4.30e-07 (0.0793 ± 0.036)	1.66e-14 (0.1797 ± 0.0893)	0.01 (0.4966 ± 0.2477)	1.80e-03 (0.4875 ± 0.1498)
Literature	Unfiltered	0.00 (0.0942 ± 0.0609)	0.00 (0.2574 ± 0.1058)	0.00 (0.5411 ± 0.2516)	0.00 (0.51819 ± 0.1421)
	Filtered	2.65e-13 (0.0593 ± 0.0290)	1.65e-11 (0.1778 ± 0.0783)	6.715e-05 (0.4436 ± 0.2243)	6.44e-06 (0.4436 ± 0.2243)

Out of the studied methods, SGT was the method yielding more significance in protein pair distance across Total and DR pair samples. Embeddings gathered by this method were already one-dimensional and did not require “lossy” mean pooling in contrast to other methods, explaining higher discernment of distance patterns. One-Hot encoding methods were second in terms of distance, possibly because they were not prone to overfitting and did not require fine-tuning and were less affected by mean pooling or training sample protein space in this regard. SeqVec CNN and biLSTM took pattern dependencies between aminoacids in two directions and BERT ProTrans included an attention mechanism for training on sequence data as well. However, both methods depended on fine-tuning on the used protein space and were likely affected by this mean pooling, but had wider distributions on protein-pair cosine distance data.

As a result of these findings, the study showcases examples of proteins that have short distance in embedding space and potentially hold promising drug repurposing opportunities or produce explainable associations between protein pairs. Examples of DR drug-disease candidates with low protein pair distance per method can be found in Table 4.

**Table 4:** Examples of disease-drug pairs yielding low protein distance for each method and potential evidence linking both terms

Embedding method	Protein A*(disease-associated)*	Protein B*(drug target)*	Disease	Drug	Distance value	Potential evidence
One-Hot	Q9NUW8 (TDP1)	P02708 (CHRNA1)	Spinocerebellar Ataxia (SCA), Autosomal Recessive with axonal neuropathy	Lamotrigine	0.0187	Lamotrigine reported benefits in relief of gait disturbance in Machado-Joseph Disease patients with early ataxia, potential suppression of pathogenic variant expression due to associated inhibition of glutamate excitatory neurotransmitter release [45]
SGT	O76082 (SLC22A5)	P50416 (CPT1A)	Renal carnitine transport defect	L - Carnitine	0.0818	Protein pairs binding the same ligand (L-Carnitine). Ligand used in an interventional study [46] on 8 adults with Primary Carnitine Defficiency, with an increase outcome on skeletal muscle fat oxidation rates during exercise. [47]
ProtBERT	Q9Y4W6 (AFG3-like protein 2)	Q92769 (HDAC 2)	Spinocerebellar Ataxia (SCA) 28	Dopamine	0.0924	Ligand with inhibitor activity on Histone Deacetilases (HDACs), suppressing apoptosis and decelerating neurodegeneration, positive effects on locomotor function in SCA disease subgroup studies (Machado-Joseph Disease) [48, 49]
SeqVec	Q9UBY8 (CLN8 protein)	P00492 (HPRT1)	Ceroid Lipofuscinosis 8 (CLN8)	Mercaptopurine	0.1554	Interventional study with ligand sharing target - Mycophenolate - reporting no adverse effects in CLN3 patients, immunosupresant potential benefits against neurodegeneration with secondary autoimmunity pathogenicity links [50].

An example of the potential DR candidates, L - Carnitine, was present for SGT embedding protein pair distance results. L-Carnitine or 3-hydroxy-4-trimethylaminobutyrate, is a quaternary amine synthesized in vivo by the liver, kidneys and brain from two essential amino acids, lysine and methionine. Carnitine is responsible for the transport of fatty acids into the mitochondria, the cellular organelles responsible for energy production [51]. The cellular uptake of carnitine in skeletal muscle, heart, kidney, lymphoblasts, and fibroblasts is mediated by Solute carrier family 22 member 5, a transporter protein encoded by SLC22A5. A deficiency in the function carried out for this protein is associated to the onset of Renal carnitine transport deficiency leading to a systemic Primary Carnitine Deficiency (PCD). In the present work, the protein has scored 0.081 SGT embedding cosine distance with the liver isoform of Carnitine O-palmitoyltransferase 1 (CPT1A), also binding L-Carnitine with palmitoyltransferase activity. This protein has the role of catalyzing the transfer of the acyl group of long-chain fatty acid-CoA conjugates onto carnitine, an essential step for the mitochondrial uptake of long-chain fatty acids and their subsequent beta-oxidation in the mitochondrion. An interventional clinical study was performed by supplementing L-Carnitine on 8 subjects with a PCD, which thereby had limited fat oxidation during exercise. The study showed that skeletal muscle fat oxidation rates during exercise were higher with L-Carnitine treatment, suggesting a potential prevention of cardiac complications in asymptomatic PCD patients by this treatment.

As another example of DR candidates for SeqVec, we have found Mercaptopurine for Neuronal (Juvenile) Ceroid Lipofuscinosis 8 (CLN8) lysosomal storage disorder. This affection is characterized by progressive vision loss, cognitive and motor dysfunction and epilepsy, has an early onset at 5-10 years of age, and results in shortened life expectancy [52]. Drugs used for the treatment of this disorders only include Antiepileptic drugs and are palliative of symptom and clinical manifestations [53]. However, an initial assessment of risk and effects of the administration of Mycophenolate on a blind-randomized trial with 19 individuals affected by Juvenile Neuronal Ceroid Lipofuscinosis was studied [50]. Mycophenolate is an immunosupresant eligible for DR, as CLN disease associated clinical and preclinical data points pathogenesis to central nervous system inflammatory response and secondary autoimmunity [54], and had a high tolerability rate for CLN patients[50]. Both Mycophenolate and the DR candidate Mercaptopurine are inhibitors of Inosine Monophosphate Dehydrogenase (IMPDH), which blocks de novo biosynthesis of purine nucleotides, a pathway lymphocyte proliferation / antibody generation is stringently dependent upon [55, 56]. In the present work, the protein associated to the disease - CLN8 protein - has scored 0.1554 SeqVec embedding cosine distance with a Hypoxanthine-guanine phosphoribosyl transferase, inhibited by Mercaptopurine and thereby unable to activate IMPDH in this pathway.

An example of a DR candidate resolved by BERT embedding protein pair distances is the indication of Valproic acid for Spinocerebellar Ataxia (SCA) 28. This new disease drug association results from embedding protein distance between AFG3-like protein to Histone deacetylase inhibitor of 0.092. Valproic acid (VPA) is a HDAC inhibitor with bipolar and seizure disorder pharmaceutical indication uses [57]. Histone deacetylation (HDAC) inhibitor VPA has been tested for safety and efficacy in several interventional studies with patients affected by another subtype of SCA (Machado-Joseph Disease), and found patients with improved locomotor function and survival time in different dosage with minor adverse effects [48, 49]. Histone acetylation levels in this affection are low and affect gene expression. These findings suggest that VPA operates on the counterbalancing and/or suppressing of apoptosis and rescuing the hypoacetylation levels of histone H3 and H4, delaying neurodegeneration [49, 58].

Another result of DR indications by One-Hot embedding distance of protein pairs is Lamotrigine (LTG) for the former disorder (SCA). This is a result of SCA disease-associated Tyrosyl-DNA phosphodiesterase 1 (TDP1) OneHot encoding matching a distance of 0.0187 with Acetylcholine receptor subunit alpha (CHRNA1). LTG is indicated for treatment of seizures and bipolar I disorder, and acts through sodium channel-mediated inhibition, suppressing the release of the excitatory neurotransmitter glutamate [45]. A former study in Machado-Joseph Disease effects of LTG reported benefits in relief of gait disturbance in patients with early ataxia, possibly linked to decreased expression of the associated pathogenic variant gene [59, 60].

It is important to note that these pairs are a small subset of examples that suggest novel target leads for treatment, from a target-screening standpoint. However, this remains an in silico prediction without experimental evidence. Predictions resulting from this method still demand testing on preclinical in vivo models or clinical trials, and do not substitute de novo drug development in any way. Ultimately, the results of this study reveal that an embedding-based approach can convey protein function and similarity, and thereby be favourable data sources narrow down the use of a vast list of target candidates, or to leverage for modelling along with other information sources (e.g. train more complex neural network models such as GNN, an approach tested within the DISNET framework).

## Conclusions

DR has emerged as an alternative to de novo drug development, which involves high costs, long times, and risks in research. In this context, DISNET’s biomedical knowledge can provide new insights into drugs and diseases to generate DR hypotheses. One of the main conclusions of the present work in the light of the obtained results is that protein sequences can play an important role in DR and could thus be used in the future to predict new candidates.

We elicited this statement in view of other secondary conclusions derived from the findings of the present study. Sequence embeddings aggregate across biological properties such as protein functional classes and number of associated diseases. Embedding sequence data techniques robustly encompass distinct patterns in DR data in comparison to the rest of protein pairs present in DISNET. We have computed distance metrics by comparing embeddings of DR protein pairs with the rest of DISNET samples. They have consistently yielded significant differences across distinct methods and in unfiltered and filtered by protein class subsets.

The outcomes of this study open valuable prospects in the use of sequence data that narrow the molecular space of new unrealized indications for diseases. However, some limitations were found. The large volume of data was a challenge in the study, resulting in computation difficulties involved in data handling that reached more than 171 million protein pairs for distance search. Moreover, given these data amounts, a biological interpretation and validation of the DR protein pairs was unfeasible. On another note, we have not studied the role of tridimensional structural data, which could provide complementary relevant information to the task of DR.

Future lines of this study would include covering other sources in the context of a disease network to generate the hypothesis of DR. As an example, a study of gene sequence data could be undertaken in the context of DR. In addition, there is the possibility of enriching the knowledge associated to the binding of drugs with embeddings using the encodings of the drugs based on their SMILES codes. Also, other metrics of distance between embeddings could be studied, such as Jaccard or Dice indexes. Along these lines, the next fundamental step would be to leverage sequence data representations jointly with other DISNET sources. As the project aims to generate prediction links in the disease network by means of Graph Neural Networks (GNNs), this new information could be considered for modeling; for instance, by representing the feature vectors of the protein nodes in the network with these embeddings. Moreover, in this work, we have employed unsupervised ML pretrained standards as an initial approach. However, it would be recommendable to train these models not using the entire universe of proteins and targeting better the challenge at hand, that is, understanding repurposing processes.

### Supplementary Information

Supplementary Material 1.

## Data Availability

The dataset supporting the conclusions of this article is included within the article (and its additional file "data.xlsx").
