# Supplementary material for: Protein sequence analysis in the context of drug repurposing
Source: BMC Med Inform Decis Mak. 2024 May 13;24:122. doi: 10.1186/s12911-024-02531-1 (PMC11092136; doi:10.1186/s12911-024-02531-1)
Supplement: Supplementary file 1 — Supplementary Material 1. [file 12911_2024_2531_MOESM1_ESM.zip › GarciaSanchez - Supplementary Materials.pdf]

Protein sequence analysis in the context of drug repurposing

Natalia García Sánchez[1], Esther Ugarte Carro[1], Lucía Prieto Santamaría[1,2] and Alejandro Rodríguez González[1,2,*]

[1] Centro de Tecnología Biomédica

[2] ETS de Ingenieros Informáticos, Universidad Politécnica de Madrid, Boadilla del Monte, Madrid, Spain,

Supplementary materials

SM Table 1. Summary of DISNET's data typology. We include entities, features, and relationships employed in the study. A description, DISNET layer, total count, the identifiers' nature, data sources and accessed date are provided. 2

SM Table 2. Characteristics of total state-of-the-art reviewed sequence embedding methods. Method characteristics, architectures, and source training data when pretraining methods are present. In addition, it presents the supervised embedding methods that were also tested, which are highlighted in blue. 3

SM Table 3. Diseases, drugs, and protein - target tuple counts for each DR case subset. The final characteristics of the total protein target pair number in DISNET group was given by also discarding proteins that had identical identifiers with their targets. 4

SM Figure 1. Cosine distance distributions retrieved for the protein pairs participating in the filtered DR cases. We depict the distributions of the cosine distance computed between the embeddings provided by each of the four studied methods and in terms of boxplots. 5

SM Table 1. Summary of DISNET's data typology. We include entities, features, and relationships employed in the study. A description, DISNET layer, total count, the identifiers' nature, data sources and accessed date are provided.

		Description	DISNET layer	Count	Identifiers	Sources	Access date
ENTITIES	**Diseases**	Data representing diseases	Phenotypical	15,415	DISNET own identifiers	Wikipedia Mayo Clinic PubMed	February 2018 – September 2022 (twice a month)
			Biological	24,314	UMLS CUIs	DisGeNET	May 2020
			Drugs	8,366	UMLS CUIs	CTD	May 2020
	**Genes**	Data representing genes	Biological	20,610	NCBI identifiers	DisGeNET	May 2020
	**Proteins and Targets**	Data representing proteins and drug targets	Biological	18,521	UniProt Accession Numbers	UniProt	May 2020
			Drugs	13,382	ChEMBL identifiers	ChEMBL	May 2020
	**Drugs**	Data representing drugs of different molecular types	Drugs	3,944	ChEMBL identifiers	ChEMBL	May 2020
				2,540	DrugBank identifiers	DrugBank	May 2020
FEATURES	**Protein sequences**	Data representing the sequences of the proteins	Biological	18,520	String of amino acids abbreviations	NCBI	January 2022
	**Protein classes**	Data representing PantherDB protein classes	Biological	29	PantherDB	DisGeNET	May 2020
RELATIONSHIPS	**Disease - Gene**	Associations between diseases and their related genes	Biological	358,209	-	DisGeNET	May 2020
	**Gene - Protein**	Associations between genes and the proteins they encode	Biological	15,770	-	DisGeNET	May 2020
	**Drug - Disease**	Associations between diseases that are indications for drugs and drugs	Drugs	1,218,607	-	CTD	May 2020
	**Drug - Target**	Associations between targets to which drugs are directed and drugs	Drugs	7902	-	ChEMBL	May 2020
						DrugBank	May 2020

SM Table 2. Characteristics of total state-of-the-art reviewed sequence embedding methods. Method characteristics, architectures, and source training data when pretraining methods are present. In addition, it presents the supervised embedding methods that were also tested, which are highlighted in blue.

	**One-Hot Encoder**	**Sequence Graph Transform** (2021) *Ranjan et al.*	**BioVec (word2vec)** (2015) *Asgari et al*.	**SeqVec** (2019) *Heinzinger et al.*	**BERT ProTrans** (2021) *Ahmed Elnaggar et al.*	**tBepler** (2020) *Bepler and Berger*	**ESM-1b** (2020) *Alexander Rives et al.*
**Method**	Binary encoding	Distance encoding	Skip gram ML autoregressive encoding	DL bidirectional contextual encoding	Deep masked contextual encoding	Deep bidirectional contextual encoding	Deep masked contextual encoding
**Input**	Sequence	Sequence	One hot 3-kmers	Encoded	Encoded	One hot	Encoded
**Output**	1 Tensor	1 Feature vector	3 vector Tensor (1 per 3-mer)	3 Tensors (1 per layer)	1 Tensor	3 Tensors (1 per layer)	1 Tensor
**Embedding feature vector size**	(Sequence length x 21)	441	100	(Sequence length x 1,024)	(Sequence length x 1,024)	(Sequence length x 121)	(Sequence length x 6,024)
**Implementation libraries**	-	sgt package	Gensim / Tensorflow	Pytorch / AllenNLP	Pytorch / Tensorflow	Pytorch	Pytorch
**Architecture**	-	-	Skip gram Feed Forward Network (FNN)	biLSTM (Recurrent RNN)	Transformer	biLSTM (Recurrent RNN)	Transformer
**Layers (nodes)**	-	-	1	1 CNN (1024) + 2 biLSTM (1,024 nodes each)	30 layers of biLSTM stacked encoders	3 biLSTM (512 nodes each)	34 layers of biLSTM stacked encoders
**Supervision**	-	-	-	-	Available on structural localization supervision	Structural self-supervision	Available Multiple Sequence Alignment (MSA) supervision
**Pretrained models**	-	-	Yes	Yes	Yes	Yes	Yes
**Databases used in pretraining**			Reviewed Swiss-Prot (0.5 M sequences)	UniRef50 (33M sequences)		UniRef90 (100 M sequences)	UniRef50

SM Table 3. Diseases, drugs, and protein - target tuple counts for each DR case subset. The final characteristics of the total protein target pair number in DISNET group was given by also discarding proteins that had identical identifiers with their targets.

Source of information	Subset	Diseases	Drugs	Disease associated proteins	Target proteins	Total Protein Target pairs
**RepoDB**	Unfiltered	119	35	7,315	122	201,250
	Filtered	22	17	48	27	51
**Literature Repurposed Diseases**	Unfiltered	58	39	7,224	127	113,379
	Filtered	18	11	39	17	43
**DISNET**		8,366	3,944	8,834	1,162	**171,245,271**

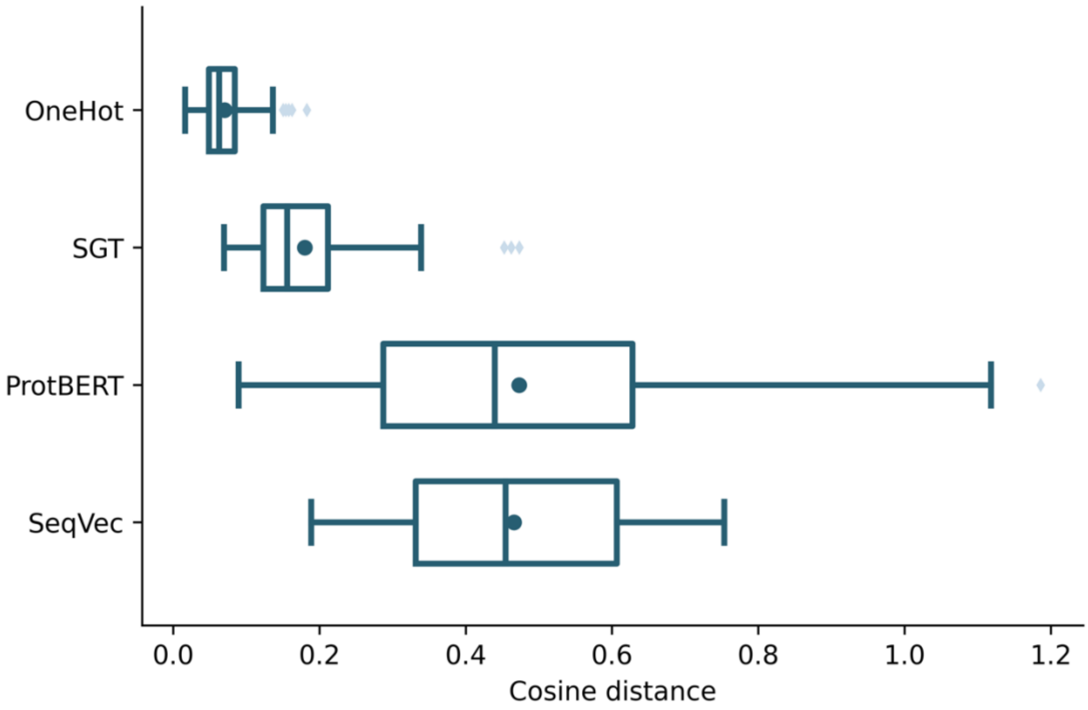

SM Figure 1. Cosine distance distributions retrieved for the protein pairs participating in the filtered DR cases. We depict the distributions of the cosine distance computed between the embeddings provided by each of the four studied methods and in terms of boxplots.

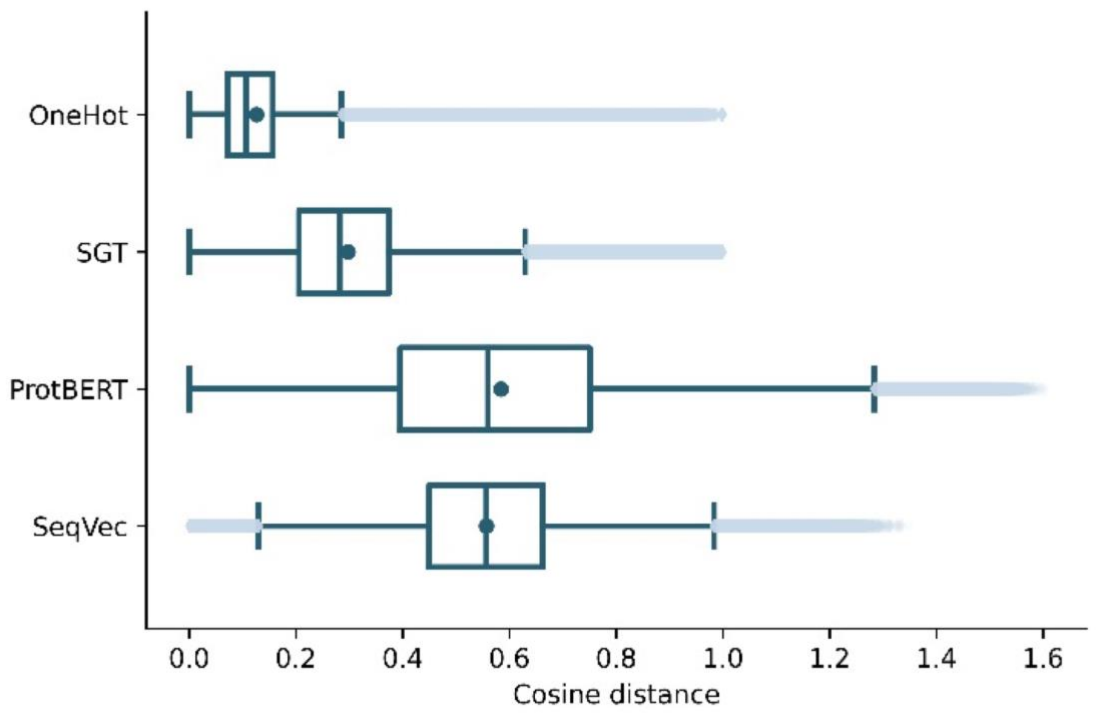

SM Figure 2. Cosine distance distributions retrieved for all possible protein pairs in DISNET. We depict the distributions of the cosine distance computed between the embeddings provided by each of the four studied methods.

Additional “data.xlsx” description

Excel file with different sheets that contain the data used in the analysis.

- **“DR cases – repoDB”**

Drug repurposing cases extracted from the repoDB database. We excluded those cases where the disease and the drug shared the drug target protein. The GDA score was depicted too.

- **“DR cases – Literature”**

Drug repurposing cases selected from the Literature[1]. We excluded those cases where the disease and the drug shared the drug target protein. Moreover, we only considered the new disease for which the drug was repositioned and not the original one for which it was indicated. Cases where the disease and the drug shared the target protein were excluded. The GDA score was depicted too.

- **“PP – repoDB”**

Unique protein pairs from the drug repurposing repoDB cases.

- **“PP – Literature”**

Unique protein pairs from the drug repurposing literature cases.

- **“PP by class – repoDB”**

Protein pairs filtered by PANTHERdb class from the drug repurposing repoDB cases. We made sure the pairs did not share class or classes.

- **“PP by class – Literature”**

Protein pairs filtered by PANTHERdb class from the drug repurposing literature cases. We made sure the pairs did not share class or classes.

- **“PP – Distances”**

The distance value for each protein pair is included for every embedding method. We indicated if the protein pair belonged to repoDB, Literature or both datasets.

- **“PP by class – Distances”**

The distance value for each protein pair filtered by PANTHERdb class is included for every embedding method. We indicated if the protein pair belonged to repoDB, Literature or both datasets.

- **“PP by class – means flags”**

We included the same dataset as the one specified in the previous sheet, with the same information but with the names and symbols for both proteins and the names for the drug and the disease, too. The score of the association (GDA) between the new disease and the protein to which it is related has been also included. Moreover, the 4 last columns include a flag for each of the embedding methods that represent whether the distance value is below the mean (1; they are more similar) or not (0; they are less similar). The mean stands for the average value of the distance computed for every pair of proteins according to each embedding method. Protein pairs showing distance values below the mean for each of the 4 embedding methods are coloured in green.

References

1. Prieto Santamaría, L. *et al.* A data-driven methodology towards evaluating the potential of drug repurposing hypotheses. *Comput. Struct. Biotechnol. J.* **19**, 4559–4573 (2021).
